# Control of the COVID-19 pandemic is derailing the fight against typhoid, dengue, and measles in Pakistan

**DOI:** 10.7189/jogh.12.03040

**Published:** 2022-07-16

**Authors:** Muhammad S Rana, Muhammad Usman, Khalid J Alzahrani, Muhammad Masroor Alam, Aamer Ikram, Muhammad Salman, Massab Umair

**Affiliations:** 1 National Institute of Health, Islamabad, Pakistan; 2 Department of Clinical Laboratories Sciences, College of Applied Medical Sciences, Taif University, Taif, Saudi Arabia

Infectious diseases have repeatedly reshaped the course of civilization, resulting in significant morbidity and mortality around the world. Infectious diseases respect neither borders nor barriers and 70% of the world is underprepared to prevent, detect, and respond to them quickly and effectively. [1].

SARS-CoV-2 is the example of the latest emerging pathogen resulting in over 426 million cases including 5.9 million deaths across the globe [2]. Similarly, in Pakistan, the number of confirmed cases reached 1 502 641 including 30 053 deaths as of February 22, 2022 [2]. We review and discuss the unprecedented upsurge in some of the notifiable diseases to highlight how COVID-19 has derailed the fight against multiple infectious diseases including typhoid, dengue, and Measles in Pakistan.

The crushing demands of the COVID-19 pandemic have strained health care systems and capacity around the world. Many communities have been responding to the pandemic and grappling with the collateral damage of COVID-19 on other public health crises including infectious diseases.

Compared with other countries around the world, Pakistan has had a relatively mild experience with the COVID-19 pandemic. On the other hand, Pakistan is endemic to multiple infectious diseases including typhoid, dengue, and Measles, which were already on the rise and have been compounded by the COVID-19 pandemic. Due to the disruption of immunization and other essential services for infectious diseases including Typhoid, Measles, and dengue the un precedential rise in cases was reported in 2021 in Pakistan. As of December 2021, over 174 000 typhoid cases including 15 deaths have been reported in the country as compared to 22 000 cases reported from 2016 to 2020 [3]. Similarly, 60 762 dengue cases including 237 deaths have been reported in 2021 compared to 3442 cases reported in 2020 [4]. Despite the availability of a safe and effective vaccine, measles remains one of the leading causes of morbidity and mortality among children in Pakistan and, an unprecedented rise in Measles outbreaks was reported with over 28 215 suspected measles cases including 800 measles-related deaths in 2021 [5] as compared to 6000 cases including 53 deaths reported in 2020. A comparison of reported typhoid, dengue, and Measles cases reported during 2020 and 2021 is presented in **Table 1**. The entire COVID-19 pandemic put a lot of strain on the immunization, surveillance, and vector control measures in developing countries including Pakistan.

**Table 1:** Comparative analysis of typhoid, dengue, and measles cases reported during 2020-2021

Disease	2020	2021	% change
**Typhoid**			
Total cases	22 000	174 000	87.30%
Deaths	0	15	100%
**Dengue**			
Total cases	6135	60 762	90%
Deaths	0	227	100%
**Measles**			
Total cases	6485	20 313	68.1%
Deaths	51	800	93.6%

Currently, Pakistan is facing four (COVID-19, typhoid, dengue, and Measles) of the biggest public health crises in its history. It is widely accepted that the COVID-19 pandemic is putting a great burden on the strength, capacity as well as capability of the health care system across the globe by demanding financial, laboratory, and trained human resources. Multiple vaccines against COVID-19 are available on the markets but there are worries that the emergence of variants of concern could escape the immunity conferred by the previous COVID-19 infection as well as by the available vaccines. Pakistan is the first county in the world to introduce the typhoid conjugate vaccine in the routine immunization schedule in 2019 [6]. Although the vaccine against dengue is available, Pakistan still has not yet started the dengue vaccination program. Due to the poor vector control measures dengue has become a leading cause of morbidity and mortality in the current years. On the other hand, the first and second doses of measles-containing vaccine were included in the expanded program on immunization schedule in 1974 and 2009 respectively. Unfortunately, Pakistan is still far below the coverage of >95% required for herd immunity, despite using the vaccines for the last fifty years. Uncountable hurdles hamper the immunization coverage in Pakistan including poor investment in public health, vaccine hesitancy, poverty, high population density, misinformation, lack of access to health care services, and lack of education. The COVID-19 pandemic has hurt the fight against other deadly infectious diseases including typhoid, dengue and measles, HIV, malaria, diphtheria, pertussis, and tuberculosis. In Pakistan, the knock-on effects on typhoid, dengue and measles, and other vaccine-preventable diseases could exceed the direct impact of COVID-19.

To successfully tackle multiple outbreaks, focused interventions such as proper investment in public health, coverage of routine immunization, stringent vector control measures, active laboratory testing, patient isolation, availability of diagnostic kits, awareness campaigns through electronic, print, and social media, vaccination against hesitancy, timely response, stainable strategies are urgently needed.

**Figure:**
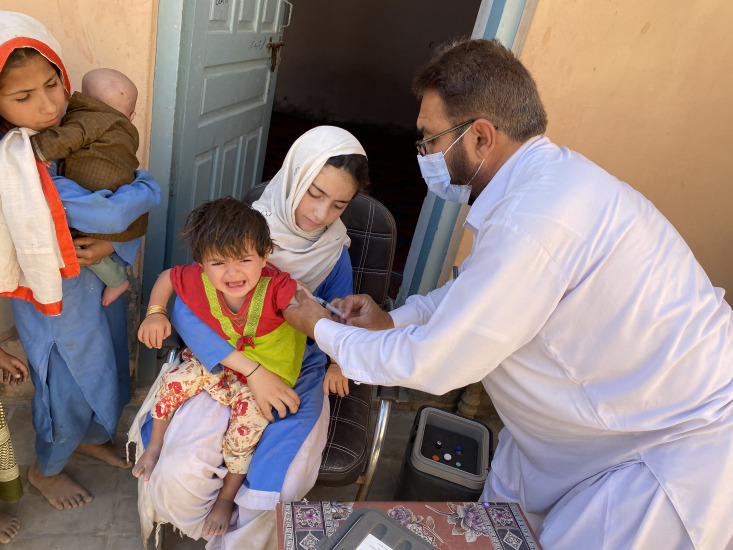
PHOTO: Millions of children are at risk of missing routine vaccination in Pakistan (from the author’s own collection, used with permission).

Health authorities and policymakers, especially those in high-risk areas of Pakistan, should fully consider the burden of other infectious diseases in the era of the COVID-19 pandemic by developing sustained policies with a focus on the control, prevention, and elimination of other infectious diseases.

Otherwise, the COVID-19 pandemic could derail promising control and elimination efforts for typhoid, dengue, and measles in Pakistan. If the stringent measures are not taken on urgent bases, the derailment of the other infectious diseases control and elimination due to COVID-19 represents a new urgency for Pakistan and its impact will be behind the borders.
